# 791 Retrospective Review of Historical Data to Determine Outcomes in Patients with Necrotizing Soft Tissue Infections

**DOI:** 10.1093/jbcr/irae036.332

**Published:** 2024-04-17

**Authors:** Austin Price, Rajiv Sood, Zaheed Hassan, Shawn P Fagan, Kade Hardy, Beretta C Coffman, Bounthavy F Homsombath

**Affiliations:** JMS Research Foundation, Inc., Augusta, GA; JMS Burn Center at Doctors Hospital Augusta, Augusta, GA; JMS Burn Center at Doctors Hospital, Augusta, GA; Joseph M. Still Research Foundation, Inc., Augusta, GA; JMS Research Foundation, Inc., Augusta, GA; JMS Burn Center at Doctors Hospital Augusta, Augusta, GA; JMS Burn Center at Doctors Hospital, Augusta, GA; Joseph M. Still Research Foundation, Inc., Augusta, GA; JMS Research Foundation, Inc., Augusta, GA; JMS Burn Center at Doctors Hospital Augusta, Augusta, GA; JMS Burn Center at Doctors Hospital, Augusta, GA; Joseph M. Still Research Foundation, Inc., Augusta, GA; JMS Research Foundation, Inc., Augusta, GA; JMS Burn Center at Doctors Hospital Augusta, Augusta, GA; JMS Burn Center at Doctors Hospital, Augusta, GA; Joseph M. Still Research Foundation, Inc., Augusta, GA; JMS Research Foundation, Inc., Augusta, GA; JMS Burn Center at Doctors Hospital Augusta, Augusta, GA; JMS Burn Center at Doctors Hospital, Augusta, GA; Joseph M. Still Research Foundation, Inc., Augusta, GA; JMS Research Foundation, Inc., Augusta, GA; JMS Burn Center at Doctors Hospital Augusta, Augusta, GA; JMS Burn Center at Doctors Hospital, Augusta, GA; Joseph M. Still Research Foundation, Inc., Augusta, GA; JMS Research Foundation, Inc., Augusta, GA; JMS Burn Center at Doctors Hospital Augusta, Augusta, GA; JMS Burn Center at Doctors Hospital, Augusta, GA; Joseph M. Still Research Foundation, Inc., Augusta, GA

## Abstract

**Introduction:**

Necrotizing soft tissue infections are rare (about 0.3-15 cases per 100,000) but rapidly progressive, life-threatening bacterial infections that can destroy the epidermis, dermis, subcutaneous tissue, fascia, and muscle (Chen, 2020). The mortality rate is 30-90% (van Stigt, 2021; Wallace, 2021). Diagnosis requires surgical debridement to confirm. Risk factors are used to create the Laboratory Risk Indicator for Necrotizing Fasciitis (LRINEC) (Wong, 2004). The validity of this tool has come into question; more recently, a study in the Netherlands attempted to identify other prognostic factors to supplement this tool (van Stigt, 2021). This study compares the mortality rate among patients diagnosed with NSTI to what is reported in the literature.

**Methods:**

This is the initial analysis for a large retrospective review of approximately 650 patients. The total n for this report is 101, data collection remains ongoing.

**Results:**

In this data set, there were 70 males and 31 females. The average age was 51. 83% lived and 17% died. This mortality rate is lower than the reported 30%-90% in the literature. Fifty-six patients (55%) were admitted to the ICU; of these, 27% died. Forty-five patients (45%) were admitted in a non-ICU setting, and 4% died. Of those who lived, the mean area affected was 688.9 cm2; for those who died, the mean area affected was 1588.9 cm2 (p=0.027795). Average LRINEC scores were 7 in both groups. Disposition was notable in that 62% of patients who lived were discharged to home, 13% to rehabilitation, and 8% to another facility.

There were significant differences in the incidence rate of Sepsis (p= < 0.00001), Renal Failure (p= < 0.00001), and Respiratory Failure (p= < 0.00001) between the two groups. There was also a significant difference in the use of NPWT between the two groups (p=0.007889). There was a significant difference in the Hgb A1c values between the two groups (p=0.039852), but, unexpectedly, the Lived group had a higher average Hgb A1c. There was a significant difference in the Lactate values between the two groups (p=0.002921).

**Conclusions:**

In 26% of patients treated and confirmed by surgical excision to have an active necrotizing process, there was an LRINEC score of less than 6. LRINEC may not be the best diagnostic tool to measure active necrotizing processes. Outcomes in our unit were not affected by the location of the affected areas. Factors impacting mortality seem to be related to ICU sequelae. Further analysis of the complete data set should establish stronger significance to these findings.

**Applicability of Research to Practice:**

It is beneficial to caregivers to disseminate and share outcomes information, especially related to unique clinical indications such as Necrotizing Skin and Soft Tissue Infections. Our data also contributes to data related to LRINEC scores and their applicability as a diagnostic indicator of this process. In 28% of our patients, the score would not have indicated that the wound was necrotizing, but the surgical excision did.

